# PIPAC for Gastrointestinal Malignancies

**DOI:** 10.3390/jcm12216799

**Published:** 2023-10-27

**Authors:** Sara K. Daniel, Beatrice J. Sun, Byrne Lee

**Affiliations:** Department of Surgery, Stanford University, Stanford, CA 94305, USA

**Keywords:** peritoneal metastases, intraperitoneal chemotherapy, PIPAC

## Abstract

The peritoneum is a common site of metastases for gastrointestinal tumors that predicts a poor outcome. In addition to decreased survival, peritoneal metastases (PMs) can significantly impact quality of life from the resulting ascites and bowel obstructions. The peritoneum has been a target for regional therapies due to the unique properties of the blood–peritoneum barrier. Cytoreductive surgery (CRS) and heated intraperitoneal chemotherapy (HIPEC) have become accepted treatments for limited-volume peritoneal disease in appendiceal, ovarian, and colorectal malignancies, but there are limitations. Pressurized intraperitoneal aerosolized chemotherapy (PIPAC) improves drug distribution and tissue penetration, allowing for a minimally invasive application for patients who are not CRS/HIPEC candidates based on high disease burden. PIPAC is an emerging treatment that may convert the patient to resectable disease, and may increase survival without major morbidity, as indicated by many small studies. In this review, we discuss the rationale and benefits of PIPAC, as well as sentinel papers describing its application for gastric, colorectal, appendiceal, and pancreatobiliary PMs. While no PIPAC device has yet met FDA approval, we discuss next steps needed to incorporate PIPAC into neoadjuvant/adjuvant treatment paradigms, as well as palliative settings. Data on active clinical trials using PIPAC are provided.

## 1. Introduction

The peritoneum is a common site of regional spread in advanced-stage gastrointestinal cancers, including gastric, appendiceal, and colorectal adenocarcinoma. Compared to lung or liver metastases, peritoneal metastasis (PM) is associated with worse long-term outcomes for many tumor types despite advances in systemic chemotherapy [1,2]. Additionally, bowel obstructions and ascites from PM have significant negative impacts on the patient’s quality of life [3]. Systemic chemotherapy has not been successful overall at palliating symptoms of PMs, but researchers have been studying direct intraperitoneal therapies for over 60 years. Many reasons for the relative resistance of PMs to systemic chemotherapy have been explored, including overall poor peritoneal vascularization and the mesothelial-to-mesenchymal transition of peritoneal fibroblasts leading to changes in extracellular architecture [4,5,6]. Inspired by peritoneal dialysis, it was discovered that the hydrophilic nature and size of many anti-cancer drugs prevent absorption through the peritoneum and allow larger drug concentrations to accumulate in the peritoneal cavity compared to plasma [7,8]. Additionally, hyperthermia was found to kill tumor cells in animal models, resulting in the most common form of intraperitoneal (IP) chemotherapy today, heated intraperitoneal chemotherapy (HIPEC) [9,10]. The modern application of HIPEC involves a pump machine that helps circulate heated chemotherapy through tubing and cannulae temporarily sewn into the abdominal cavity, as described previously [10,11]. Normothermic IP chemotherapy (NIPEC) has also been used with chemotherapeutic agents without significant synergistic effects with heat, such as paclitaxel. NIPEC is administered with an indwelling catheter, either with or without cytoreduction. It can also be administered repeatedly, often in the outpatient setting, which makes it ideal for patients who may never be candidates for cytoreductive surgery (CRS) [12].

Peritoneal concentration and heat are not the only determinants of IP chemotherapy effectiveness; however, the drug must also penetrate into cancerous tissue and enter tumor cells. Some of the limitations of HIPEC/NIPEC include the need to agitate the abdomen during perfusion, the dependent nature of the fluid, and the importance of an adequate cytoreduction to ensure the intraperitoneal chemotherapy penetrates the desired tissue [10]. Adequate cytoreduction, resecting all disease larger than 2.5 mm (Completeness of Cytoreduction (CC) score of 0–1), is necessary to allow for effectiveness of the IP chemotherapeutic agent [13,14]. Pressurized intraperitoneal aerosolized chemotherapy (PIPAC) was designed to address some of the limitations. Pre-clinical studies have shown that the aerosolized drug particles in PIPAC lead to a more evenly distributed drug delivery within the peritoneal cavity than the liquid form in HIPEC [15,16]. Additional proposed benefits for PIPAC include the pressure of the capnoperitoneum allowing for better tumor penetration even in the absence of cytoreduction [17,18,19]. The procedure is performed in a minimally invasive (MIS) fashion, which would allow for a shorter hospital length of stay (LOS), reduced pain, and otherwise improved quality of life (QOL), while having the ability to continue systemic chemotherapy [20,21]. The first case series for PIPAC was published in 2012, and in this review, we will summarize the 10 years of evidence regarding the potential benefits and risks of PIPAC compared to HIPEC and NIPEC [15]. Additionally, we will discuss the outcomes for PIPAC trials for treatment of gastric, colorectal, appendiceal, and pancreatobiliary tumors, as well as discuss future directions for PIPAC application.

### 1.1. Surgical Technique

**Figure 1:**
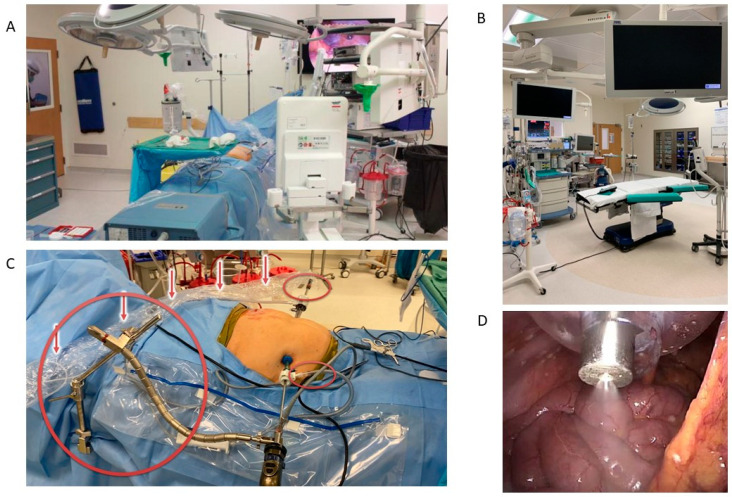
PIPAC OR set up. These intraoperative pictures demonstrate the configuration of monitors from (**A**) inside the OR and (**B**) inside the antechamber where staff are located during aerosolization. In the two-port system displayed in (**C**), a self-retained camera holder system is used, and the suction port is connected to the camera port, while the capnopen is in the other port (circled). The tubing connecting the capnopen to the power injector is indicated by arrows. The resulting fine mist expected from a well-positioned capnopen is shown in (**D**). These pictures have been provided by Dr. Mustafa Raoof and Dr. Thahn Dellinger at City of Hope.

The technical aspects of PIPAC device set up, laparoscopic trocar selection, and occupational safety measures have been described in detail [22]. Briefly, access to the abdomen is obtained generally in the left upper paramedian space to best obtain a 45-degree angle to the underlying peritoneum. Both multiport and single-port access have been safely documented for PIPAC; however, two ports are most common [23]. Placement of fascial closing sutures is performed before inserting a 12 mm balloon trocar. After insufflation to 12 mm Hg, an additional 5 mm balloon trocar is placed under direct visualization to aid with the staging laparoscopy and obtaining tissue samples, in four regions. Unnecessary personnel are asked to leave the OR, and chemotherapy protective measures are worn. Safety protocols, including draping of the floor and monitors, chemotherapy waste bins, and tight connections on all tubing, should be performed. The power injector should be set to a volume flow of 30 mL/min and maximum operating pressure of 200 psi. The chemotherapy injection is started remotely and monitored on the video screen. Generally, the aerosol is left to dwell for 30 min per chemotherapeutic agent, although there have been some agents combined into a single syringe. After ensuring again that the appropriate aerosol waste line is connected, the nozzle is disposed of in the appropriate bin, and the gas is evacuated. Care must be taken to ensure appropriate fascial and subcutaneous closure. Please see Figure 1 for representative images.

Appropriate patient selection is crucial in performing PIPAC successfully. Absolute contraindications include bowel or urinary obstruction without the ability to safely and easily bypass or divert; inability to access the abdomen safely; significant abdominal adhesions; recent bowel perforation or intra-abdominal sepsis; inability to tolerate laparoscopy from a cardiopulmonary perspective; or other medical conditions that would preclude surgery, such as poor performance status [22]. While cytoreduction is generally not performed with PIPAC, one study added other surgical procedures, such as adhesiolysis, omentectomy, hernia repair, and gastrectomy, to the treatment and found no increase in surgical complications compared to PIPAC alone, although LOS, surgery time, and medical complications, such as pain, nausea, and ileus, were increased [24]. Failure to access the abdomen can be secondary to adhesions from prior procedures or from tumor involving the abdominal wall and preventing safe entry and/or expansion. The rate of intra-abdominal access failure in the literature has ranged from 0 to 22%, and this has been shown to increase with the number of previous abdominal surgeries [25,26]. It is also recommended that the use of PIPAC be limited to patients with no extraperitoneal disease or distant disease that is responding to systemic therapy.

Optimizing drug delivery for PIPAC compared to HIPEC/NIPEC must consider the aerosolization of the chemotherapeutic agent, uptake of the drug into the peritoneal nodules, and clearance of the drug from the tissue [27]. Normal healthy peritoneum has a glycocalyx covering the mesothelial layer, which overlies the interstitial tissue and capillaries. Metastatic involvement causes fibrotic changes, as well as an increase in interstitial pressure in the peritoneum that decreases drug uptake [27]. The aerosolized nature of PIPAC was theorized to provide more even distribution of chemotherapy compared to liquid form, but distribution is still not perfect [28,29]. Tissue penetration has been confirmed to increase with higher intra-abdominal pressure [30]. Electrostatic precipitation of the aerosol is being investigated as a method of encouraging deeper tissue penetration, with less time required for treatment [31]. In addition to overcoming increased interstitial pressure, the increased intra-abdominal pressure from CO_2_ insufflation causes collapse of the splanchnic vasculature, which keeps more drug in the peritoneum instead of absorbed to systemic circulation [27].

### 1.2. Drug Selection

The initial drugs tested for PIPAC delivery were oxaliplatin or a combination of cisplatin and doxorubicin; these chemotherapies had been previously selected for HIPEC due to their good systemic tolerance, synergy with heat, and lack of cell cycle specificity [17,18]. Dose escalation studies in PIPAC have been performed for cisplatin (up to 30 mg/m^2^), doxorubicin (up to 6 mg/m^2^), and oxaliplatin (up to 135 mg/m^2^); however, most clinical trials still use more arbitrary determined doses [32,33]. There have been multiple reports of increased pain after PIPAC with oxaliplatin compared to cisplatin and doxorubicin. In a study of 41 patients, 28% and 80% of the PIPACs were performed in the outpatient setting at PIPAC 1 and 3, respectively, for cisplatin and doxorubicin compared to 11% and 20% for oxaliplatin [34]. There were no readmissions after outpatient PIPAC, but post-operative opioid use was more frequent in the oxaliplatin group [34]. Another study found that pain is greatest in the first 32 h after PIPAC, with oxaliplatin more likely to be associated with moderate-to-severe pain than cisplatin and doxorubicin [35]. One rare side effect of PIPAC, only seen with oxaliplatin, is severe peritoneal sclerosis, which leads to abdominal pain and constipation/obstruction [36]. With several studies, such as PRODIGE 7, calling into question the effectiveness of oxaliplatin in HIPEC for colorectal PMs, there has been a renewed interest in other agents [37]. Mitomycin C, a drug that causes DNA cross-linking and inhibits transcription, has positive results in HIPEC for colorectal cancer, with improved locoregional control [38]. While it has not yet shown great effect in in vitro cell line studies, Mitomycin PIPAC has been used in a small number of humans with colorectal cancer and is being examined in ongoing clinical trials [39,40,41]. Taxanes have also been identified as candidate chemotherapeutics for PIPAC. In one study evaluating PIPAC with paclitaxel alone or in combination with docetaxel for gastrointestinal or gynecologic PMs, conversion to CRS/HIPEC was high at 19%, despite an initial Peritoneal Cancer Index (PCI) score of 24 [42]. Additionally, in the 31 patients with ascites, a reduction in ascites was observed in 11 (35.4%) [42]. While dosages of oxaliplatin, cisplatin, and doxorubicin were likely lower than previously reported, as they were not body-surface area based, major complications were low (12.7%), and there were no deaths [42]. Ongoing phase I trials are investigating the use of albumin-bound paclitaxel, irinotecan, and novel immunotherapies in PIPAC, although the vast majority are based out of Europe [43,44] (Table 1).

### 1.3. Comparison of PIPAC to NIPEC and HIPEC

Compared to NIPEC delivered via a catheter or port, there are benefits and risks to PIPAC. Both PIPAC and NIPEC can be used for serial treatments in the palliative setting, as well as neoadjuvant/adjuvant for high-risk tumors. Catheter-based NIPEC is designed as an outpatient treatment once the catheter has been placed, and this can be performed by proceduralists other than surgeons [12]. Because NIPEC does not use heat or pressure to deliver chemotherapy, there is less risk of ileus or other bowel-related complications, such as anastomotic leak, compared to PIPAC [45]. With fewer bowel side effects and no new incisions, there is a theoretical decreased risk in combining systemic chemotherapy including VEGF inhibitors, such as Bevacizumab, with NIPEC, but this has not been tested [12]. In addition to wound healing, major limitations of PIPAC compared to NIPEC are the systematic requirement of operating room (OR) time and general anesthesia. One benefit of requiring repeat operative intervention, however, is that it allows for laparoscopic assessment of treatment response through calculation of the peritoneal carcinomatosis index (PCI) and the peritoneal regression grading score (PRGS) [14]. The theoretical risk of increased occupational exposure to chemotherapy drugs from aerosolization during PIPAC has not been demonstrated in studies, but appropriate evacuation of the laparoscopic gas at the conclusion of the procedure is crucial. One high-volume PIPAC center in Europe requires all surgeons and nurses who perform PIPAC procedures to undergo blood and urine analysis every 6 months, and no platinum exposure has been detected after 150 cases [22]. Another occupational health study found that while air contamination was negligible, surface contamination, particularly of power injectors and trocars, varied widely [46]. For this reason, similar to HIPEC and NIPEC, proper contact precautions are mandatory.

In comparing HIPEC to PIPAC, both require an OR and general anesthesia for the patient, although OR time for PIPAC is generally shorter after accounting time for CRS. PIPAC dwell time is only 30–60 min (one or two medications), compared to 60–120 min for HIPEC perfusion. While pressure achieved for PIPAC is beneficial for tissue penetration, there is a risk of tissue damage. In a pig model of colon anastomosis, PIPAC with cisplatin had a trend towards increased anastomotic leakage on autopsy compared to HIPEC (37.5% vs. 0%, *p* = 0.20); however, the PIPAC group had more food intake and less weight loss, suggesting a higher quality of life [45]. The concentration of the drug required for PIPAC is less than doses for HIPEC, resulting in lower absorption into the bloodstream [47]. While laparoscopic HIPEC has been described, HIPEC is traditionally performed via a laparotomy in conjunction with CRS and therefore has a prolonged hospital stay and recovery off systemic chemotherapy. The overall morbidity rate after HIPEC can be as high as 55%, depending on the extent of CRS, with infections being the most common post-operative complication [48]. LOS after HIPEC can range widely, also dependent on CRS extent and possible complications [49]. PIPAC, on the other hand, usually requires a 1-to-3-night stay in the hospital, but has been performed as an outpatient procedure in a limited group of patients [34]. Additionally, systemic chemotherapy can be continued uninterrupted with PIPAC, depending on the regimen. The shortest median time to resumption of systemic chemotherapy after PIPAC has been 6 days; however, the more typical is 14 days [22,50]. Additionally, there is evidence to support that anti-VEGF therapy may not need to be paused for PIPAC in the same traditional 6 weeks needed prior to laparotomy. Although most recommend holding bevacizumab for 4 weeks pre-PIPAC, 2 weeks has been demonstrated to be safe [51]. For these reasons, HIPEC is less suitable than PIPAC for serial procedures.

### 1.4. Complications with PIPAC

Major complications during or after PIPAC have been reported, with rates of 0 to over 62%; however, as with all procedures, this decreases with improved patient selection and experience [22,24,33,40]. Examples of complications reported after PIPAC include superficial infection, fascial dehiscence, bowel injury during access, intestinal obstruction, aspiration, and pleural effusion [22,40,42,50]. Unique severe complications, such as sclerosing peritonitis, have also been reported rarely, and only after using oxaliplatin so far [36]. It is important to note that the majority of patients included in early PIPAC trials had widely metastatic peritoneal disease, placing them at higher risk of general post-operative complications. There are also anesthetic complications to be mindful of with the increased intra-abdominal pressure, ascites-related fluid shifts, and possible allergic reactions [20,22]. Similar to HIPEC, it is important to be aware of potential drug-specific complications; in pre-clinical models, severe tubular necrosis was seen after high cisplatin doses in pigs [17]. Additionally, neutropenia, anemia, pancreatitis, transaminitis, nausea, fatigue, and abdominal pain have been reported as minor complications [32,33,52].

The appropriate timing of PIPAC and integration with systemic therapy is also debated. Most studies on PIPAC have taken place in patients with known metastatic disease and progression on systemic chemotherapy, who were determined not to be candidates for CRS/HIPEC. As being studied with HIPEC, there is an interest in the addition of prophylactic PIPAC at the time of resection of tumors at high risk for peritoneal recurrence. In one study looking at gastric cancer, there was no increase in complications with the addition of PIPAC at the time of D2 gastrectomy [52]. There is also the question of using PIPAC in the neoadjuvant setting prior to CRS/HIPEC. In one study of 406 patients undergoing PIPAC for multiple tumor types, 21 patients, or 5%, were then considered candidates for CRS/HIPEC due to treatment response [53]. Of those 21 patients, 12 were thought to have been candidates for CRS/HIPEC originally and only received one round of PIPAC. The remaining nine patients underwent CRS/HIPEC after an average of 3.5 PIPAC cycles. A CC score of 0 or 1 was achieved in 20 of the 21 patients [53]. Another study of PIPAC in all tumor types found that 8.3% of patients were able to undergo a subsequent R0 resection and HIPEC and had no recurrence at a median follow-up of 28 months [54]. The highest rate of conversion to CRS/HIPEC from PIPAC seen in larger studies was 14% after a median of three PIPAC sessions in conjunction with systemic chemotherapy [55]. The 146 patients in this study had a median PCI of 16 and a 67% recurrence-free survival at 7 months [55]. Whether neoadjuvant, adjuvant, or palliative, PIPAC is an effective method of intraperitoneal therapy for many patients.

## 2. Clinical Data

### 2.1. Gastric

Gastric adenocarcinoma, while more prevalent in Eastern populations, has an incidence of over 26,000 cases annually in the US [56]. While 5-year overall survival has doubled since the 1970s, it remains overall low compared to other tumor types at 33% [56]. Modern treatment for patients without metastatic disease includes perioperative chemotherapy containing 5-fluorouracil (5-FU) and oxaliplatin, as well as surgical resection with a D2 lymphadenectomy [57]. Recurrence rates remain high, however, and PM comprises almost half of these recurrences [58]. Early identification of recurrence has not been shown to improve survival, so alternative treatments are being sought to prevent PM [59]. HIPEC has been used for gastric cancer with PMs; however, the PCI threshold for when CRS/HIPEC is recommended in gastric cancer has generally been lower compared to other malignancies [60,61,62]. For those who are not a candidate for HIPEC, PIPAC has been studied in gastric cancer both for treatment of known metastatic disease and for prevention of PMs at the time of gastrectomy. Studies that have evaluated PIPAC solely in gastric adenocarcinoma are included in Table 2 [50,52,63,64,65,66,67,68,69,70].

The majority of PIPAC trials that included gastric cancer have used a combination of cisplatin at 7.5 mg/m^2^ and doxorubicin at 1.5 mg/m^2^. The median number of PIPAC treatments that patients received during these studies ranged from 1.5 to 3, and the median PCI for non-prophylactic studies ranged from 10.5 to 20. Most studies allowed for ongoing systemic chemotherapy, although the regimen was only specified in one study. Major morbidity after PIPAC ranged from 0 to 37.5%, and mortality ranged from 0 to 8.3%. When reported, PRGS and overall survival for patients who underwent at least two PIPAC treatments were 40 to 83% and 15 to 16 months, respectively. Complete pathologic responses were rare, but did occur in one patient each in the studies from Di Giorgio et al. and Struller et al. [50,66]. PCI was also found to not always correlate with PRGS [50]. In Tidadani et al., the addition of PIPAC produced increased overall survival from time of PM diagnosis (12.8 months vs. 9.1, *p* = 0.056) and resulted in fewer hospitalization days in the 6 months after PM diagnosis (2 days vs 11, *p* = 0.04), without any significant increase in major morbidity (*p* = 0.62) [70]. Malignant ascites is a common problem for patients with PM from gastric adenocarcinoma. In Gockel et al., 79% of patients had stable or decreased ascites volume, while in Alyami et al., 50% of patients with ascites had complete resolution [65,68]. As with systemic chemotherapy, many patients are not able to complete treatment with PIPAC for a variety of reasons. In Khomayakov et al., less than 50% of patients were able to complete the protocol of systemic XELOX with three sessions of PIPAC [63].

There has only been one clinical trial published evaluating prophylactic PIPAC after reconstruction in laparoscopic D2 gastrectomy for high-risk tumors, which was defined as those with signet ring cell histology, positive lymph nodes, T3 or higher staging, or previous positive cytology [52]. All but one patient received pre-operative systemic chemotherapy, and 15 of 21 patients received post-operative chemotherapy. One patient had positive cytology at the beginning of the operation, but no patients had positive cytology post-PIPAC; positive cytology rates post gastrectomy have been reported as high as 60% positive, despite negative cytology at the beginning of the operation. With this data, there is the suggestion that PIPAC can neutralize any cancer cells that escaped due to manipulation during the gastrectomy or were missed with original lavage. The rates of complications were not significantly higher than previously reported for laparoscopic D2 gastrectomy without PIPAC. Two patients (9.5%) had major complications, one anastomotic leak, and one duodenal stump blowout [52]. Additional patients suffered neutropenia (4.7%), prolonged ileus (4.7%), and moderate pain (42.8%). Overall, PIPAC in conjunction with gastrectomy or as an independent procedure is safe and potentially beneficial in gastric cancer.

### 2.2. Colorectal and Appendiceal

The incidence of colorectal cancer is increasing every year in the US, most recently greater than 153,000 cases a year, making it the third most common cancer type in both men and women (56 Siegel). As modern screening and chemotherapy improve, so has the 5-year overall survival rate, which is now around 65% [56]. For non-rectal cancer, treatment generally consists of upfront resection for non-metastatic disease, followed by adjuvant chemotherapy, whereas rectal cancer resection is usually preceded by neoadjuvant chemoradiation [71,72]. PMs ultimately develop in up to 9% of patients, with over 4% being present at the time of initial diagnosis [73]. HIPEC has been studied in colorectal cancer both for treatment of known metastatic disease after CRS and as a prophylactic measure for patients with tumors at high risk of developing future PMs (perforated or T4 tumors, signet ring histology, ovarian metastases, etc.). In the setting of known PMs, the addition of oxaliplatin HIPEC to CRS did not demonstrate a benefit in colorectal cancer [37]. The COLOPEC and PROPHYLOCHIP trials evaluated adjuvant HIPEC with oxaliplatin at second-look surgeries and did not demonstrate survival or local recurrence benefits [74,75]. More recently, the HIPECT4 trial demonstrated a significantly lower local recurrence rate with prophylactic mitomycin C HIPEC and at the time of colectomy, without increased morbidity [76].

Appendiceal cancer incidence can be difficult to tease out, as it is often grouped with colon cancer and may or may not include neoplasms, such as low-grade appendiceal mucinous neoplasms (LAMNs) or high-grade appendiceal mucinous neoplasms (HAMNs) [77]. LAMN and HAMN generally do not metastasize outside of the abdominal cavity, and there are no good systemic treatments. Appendiceal adenocarcinoma and appendiceal goblet cell carcinoma with neuroendocrine features are treated with systemic chemotherapy and have a worse prognosis [77]. Development of PMs is common in these lesions if there has been perforation of the appendix or rupture during resection. CRS/HIPEC has been used for PMs from appendiceal mucinous neoplasms, as well as adenocarcinoma, with improved overall survival, including repeated operations in the same patient; however, data come mostly from retrospective analyses [78,79,80,81]. Both oxaliplatin and mitomycin have been used in HIPEC for appendiceal carcinoma, and while some data suggest improved QOL with oxaliplatin compared to mitomycin, however they have similar survival outcomes [82]. With better overall survival compared to other tumor types, the repeatability and minimally invasive nature of PIPAC offers exciting opportunities for the treatment of appendiceal neoplasms.

There have been five studies published evaluating PIPAC on colorectal and/or appendiceal malignancies (Table 3) [25,83,84,85,86,87]. For colorectal and appendiceal adenocarcinoma, PIPAC has been performed predominantly with oxaliplatin at 92 mg/m^2^, sometimes in conjunction with systemic 5-FU and leucovorin. The median number of PIPAC treatments that patients underwent was two to three, and initial PCI ranged from 11 to 29. For patients that underwent at least two PIPAC sessions, PRGS showed a response in 49 to 79% of patients, and the median overall survival was 17 to 22 months. Major morbidity varied between 6% and 25%, but mortality was low from 0 to 1%. Rovers et al. evaluated 20 patients, 7 of whom had appendiceal pathology including LAMN; however, their overall survival was the lowest at 8 months [85]. In Tabchouri et al., the largest series of 102 patients, access failure was high at 22%, but the majority of patients had undergone multiple previous surgeries, including 10% with previous HIPEC. They did find improved overall survival for those who underwent three or more PIPAC procedures compared to those who underwent two or fewer PIPAC procedures (17.2 vs 8.8 months, *p* = 0.03), especially in patients with PCI greater than 12 [25]. Somashekhar et al. looked at only patients with appendiceal carcinoma from multiple institutions, including one patient who had undergone PIPAC 15 times. They did not find that undergoing PIPAC for at least three treatments was associated with significantly decreased PCI, improved PRGS, or presence of abdominal symptoms, although they did find that PCI < 11 at the first PIPAC was associated with longer survival [86]. While it did not reach significance, survival after three or more PIPACs was 22 months compared to 10 months with one or two procedures [86]. The most recent study by Raoof et al. examined 12 patients with systemic oxaliplatin-resistant appendiceal or colorectal cancer. The percentage of patients with a pathologic response (PRGS 1 or 2) after PIPAC and median OS were lower than other studies at 45% and 12 months, respectively; however, the major complication rate was low, and two patients ultimately underwent CRS/HIPEC [87]. While studies remain small, PIPAC does seem to provide a benefit for patients with PMs from colorectal and appendiceal cancers.

### 2.3. Pancreatobiliary

Pancreatic and extra-hepatic biliary cancers have an incidence in the US of over 64,000 and 12,000 cases, respectively [56]. The overall 5-year survival rate is 12% for pancreatic cancer, making it the fourth leading cause of cancer death; 11% for extrahepatic cholangiocarcinoma; and 20% for gallbladder carcinoma [56]. Treatment for these tumors generally consists of systemic chemotherapy and resection, which varies depending on the location of the tumor [88]. While liver metastases are the most common site for pancreas and biliary cancers, PMs have been shown to be associated with worse survival outcomes [89,90]. Both HIPEC and NIPEC have been explored for pancreatic adenocarcinoma in the setting of prophylaxis after an R0 resection, as well as in the setting of CRS for limited-volume pancreatic disease [91,92,93,94,95,96]. Although early results have demonstrated a benefit, there has not been widespread adoption of CRS/HIPEC for pancreatobiliary cancers.

Table 4 summarizes the four studies evaluating PIPAC solely in pancreatobiliary malignancy [97,98,99,100]. The median number of PIPAC treatments was 2–3, and the median initial PCI when reported was 18–19. PRGS of 1 or 2 in those who underwent two or more PIPAC treatments was 50–80%, and overall survival ranged from 2.8 to 10 months. Of note, patients in the study with the poorest survival did not receive concurrent systemic chemotherapy [98]. There was no major morbidity or mortality reported in any of the studies. In studies that evaluated multiple tumor types, survival was worse for gallbladder cancer and pancreatic adenocarcinoma than extra-hepatic cholangiocarcinoma in patients eligible for PIPAC [98,99]. Additionally, PRGS and OS were better with cisplatin and doxorubicin than oxaliplatin, although the numbers are small [99]. Regarding ascites, Di Giorgo et al. found that those who had high-volume ascites (>2 L) were unlikely to be candidate for more than one cycle of PIPAC due to rapid clinical deterioration. Of the five patients who received two or more PIPAC treatments, ascites decreased or remained minimal in 60% [99]. Overall, PIPAC for pancreatobiliary PMs was found to be safe and has the potential to prolong survival in carefully selected patients.

## 3. Future Directions

While PIPAC is a promising therapeutic avenue for PMs, there are several issues that need to be further investigated prior to widespread adoption. Evaluating quality of life after PIPAC has shown no significant changes in small retrospective studies for multiple tumor types [101]. In fact, QOL tended to improve with subsequent additional PIPAC, although this difference was not significant [66]. Additionally, the PCI score as a representative of tumor burden did not influence change in the QOL after PIPAC [101]. The most common side effects reported for most patients were nausea/vomiting, fatigue, constipation, abdominal pain, and hematologic toxicities [102]. Patient-reported outcomes seem to decrease the most in the first week after the first PIPAC but are not impacted as severely with subsequent procedures [103,104]. Additionally, by 4 weeks after the third treatment, most metrics were back to baseline. Appetite loss remained the only symptom that increased throughout treatment [104]. As increased visceral adipose tissue and lower-quality muscle correlated with lower PRGS scores after PIPAC, optimizing nutrition will be important for maximizing survival after PIPAC [105]. Further work needs to be conducted on optimizing PIPAC for symptom management and determining long-term QOL effects.

The cost of PIPAC needs to be studied further, especially in the US healthcare system, if adoption is to be encouraged. Small studies out of Europe suggest a loss to the hospital, but LOS was over 3 days on average, and some costs were not covered by insurance [106]. A study in the UK looked at the cost of the addition of PIPAC with cisplatin and doxorubicin to systemic first-line chemotherapy (XELOX) or the use of PIPAC alone as a second-line treatment compared to ramucirumab [107]. The addition of PIPAC to systemic XELOX led to an increase of 0.46 in quality-adjusted life-years (QALYs), but did cost an additional GBP 31,868 per QALY. For the second-line therapy, the use of PIPAC led to an increase of 0.19 in QALYs over ramucirumab and actually saved GBP 21,474 [107]. Streamlining the PIPAC process will hopefully reduce costs and encourage this therapy to be available to more patients.

It is important to note that no PIPAC nebulizer device has yet received full FDA approval, so most of these data come from trials in Europe and Asia. Additionally, as new nebulizer devices come on to the market, it will be important to ensure equal device safety and outcomes. There has also been research into the addition of electrostatic precipitation to PIPAC [108]. As discussed earlier, we anticipate that clinical trials with higher doses of medications and different chemotherapeutic agents will be on the horizon, potentially with optimization based on each patient’s specific mutation [100]. Finally, a head-to-head comparison of safety and efficacy between HIPEC and PIPAC, both for MIS and open post-CRS applications, should be pursued.

## 4. Conclusions

PIPAC is an exciting new potential therapeutic option in both the neoadjuvant and palliative setting for patients with PMs. While there is still work being conducted to better identify patients who will benefit, an additional method to directly deliver chemotherapy to the peritoneum without the morbidity of a major surgery remains promising. Additionally, the increased tissue penetration from the pressure will allow an increased choice of chemotherapeutics to be used in PIPAC, which may open intraperitoneal treatment to an even larger group of patients. While a complete response is so far rare in response to PIPAC, longer survival with an improved QOL is a goal worthy of further research.

## Figures and Tables

**Table 1:** Ongoing PIPAC trials for gastrointestinal tumors.

Trial Number	Country	Disease Site	PIPAC Drug	Systemic Treatment
NCT05644249	Lithuania	Gastric	Cisplatin/Doxorubicin	FOLFOX
NCT05303714	Italy	Gastric	Cisplatin/Doxorubicin	FOLFOX
NCT05318794	England	Gastric	Cisplatin/Doxorubicin	Not specified
NCT04595929	Russia	Gastric	Cisplatin/Doxorubicin	FLOT
NCT03304210	Belgium	Gastric/Pancreas	Paclitaxel	Not specified
NCT04475159	Israel	Colorectal	Not specified	Not specified
NCT03280511	Denmark	Colorectal	Oxaliplatin	Not specified
NCT03868228	England	Colorectal	Oxaliplatin	Not specified
NCT04734691	Switzerland	Colorectal	Oxaliplatin	Not specified
NCT04956068	Singapore	Any	Cisplatin/Doxorubicin or Oxaliplatin	Not specified
NCT05395910	Singapore	Any	Paclitaxel	Not specified
NCT04000906	Switzerland	Any	Cisplatin/Paclitaxel	Not specified
NCT03172416	Singapore	Any	Oxaliplatin	Nivolumab
NCT05277766	Belgium	Any	Irinotecan	Not specified
NCT05431907	Israel	Any	Allocetra-OTS	Not specified
NCT04329494	USA	Any	Not specified	Not specified

**Table 2:** PIPAC for gastric cancer.

Year	First Author [Reference]	# PIPAC Patients (1/2/3/4+ Treatments (Total))	Median # Treatments	Median Initial PCI	% Previous Surgery	% Signet Ring Histology	% Extraperitoneal Disease	Concurrent Systemic Treatment Regimen	% Initial Access Failure	% PRGS Grade 1/2 w/2+ Treatments	OS (Months)	% Subsequent CRS	LOS (Days)	% Major Morbidity (CTCAE or CD 3–4)	% Mortality
2016	Khomyakov [63]	16/7/6/2 (31)	1.5	16		97%	0%	XELOX		60%	13		3	3.2%	0%
2016	Nadiradze [64]	7/7/3/7 (24)	2	16	63%	75%	17%	Not specified	4%	50%	15.4			37.5%	8.3%
2018	Gockel [65]	9/6/5/4 (24)	2	14	54%		21%	Not specified	18%		7			0%	0%
2019	Struller [66]	7/12/6/0 (25)	2	15.3	60%	88%		None		83%	6.7			12%	
2020	Di Giorgio [50]	8/13/7/0 (28)	2	20	57%	75%	21%	Not specified	4%		12.3	3.5%	2	4%	4%
2020	Ellebæk [67]	6/4/10 (20)	2.5	10.5	25%	45%		Not specified	0%	40%	4.7			10%	0%
2021	Alyami [68]	8/4/17/13 (42)	3	17		79%		Not Specified			19.1	14%	3	3%	4.7%
2022	Sindayigaya [69]	52/32/24/21 (131)	2	15				Not specified	8%	73%	10	7%	5	5%	1.4%
2022	Tidadini [70]	(17)	2	18	12%		0%	Not specified			12.8	11.8%		11.8%	0.0%
2023	Graversen [52]	(21)		0				Not specified	0%				6	9.5%	0.0%

PCI = Peritoneal cancer index, PRGS = Peritoneal regression grading score, OS = Overall survival, CRS = Cytoreductive surgery, LOS = Length of stay, CTCAE = Common terminology criteria for adverse events, CD = Clavien–Dindo.

**Table 3:** PIPAC for colorectal and appendiceal cancer.

Year	First Author [Reference]	# PIPAC Patients (1/2/3/4+ Treatments (Total))	Median # Treatments	Median Initial PCI	% Previous Surgery	% Signet Ring Histology	Concurrent Systemic Treatment Regimen	% Initial Access Failure	% PRGS Grade 1/2 w/2+ Treatments	OS after Initial PIPAC (Months)	% Subsequent CRS	% Major Morbidity (CTCAE or CD 3–4)	% Mortality
2016	Demtroder [83]	3/5/3/6 (17)	2	16	100%		Not specified	0%	79%	15.7	11.7%	23%	0%
2020	Ellebaek [84]	5/4/15/0 (24)	3	11		4.2%	Not specified	0%	67%	20.5	0%	8.3%	0%
2021	Tabchouri [25]	26/20/17/17 (102)	2	14	96%		Not specified	21.6%	74%	14		5.9%	1%
2021	Rovers [85]	4/3/7/6 (20)	3	29	55%	45%	5-FU + Leucovorin	5%	56%	8		15%	0%
2022	Somashekhar [86]	25/14/19/19 (77)	2	23	35%		Not specified	5.1%	49%				
2023	Raoof [87]	4/2/6/0 (12)	2.5	28	42%	25%	5-FU + Leucovorin	7.7%	42%	12	17%	16.7%	0%

PCI = Peritoneal cancer index, PRGS = Peritoneal regression grading score, OS = Overall survival, CRS = Cytoreductive surgery, CTCAE = Common terminology criteria for adverse events, CD = Clavien–Dindo.

**Table 4:** PIPAC for pancreatobiliary cancer.

Year	First Author [Reference]	# PIPAC Patients (1/2/3/4+ Treatments (Total))	Median # Treatments	Median Initial PCI	% Previous Surgery	Drugs/Dosage	Concurrent Systemic Treatment	% Initial Access Failure	% PRGS Grade 1/2 w/2+ Treatments	OS (Months)	% Major Morbidity (CTCAE or CD 3–4)	% Mortality
2017	Graversen [97]	0/2/3/2 (5)	3		40%	cisplatin 7.5 mg/m^2^ and doxorubicin 1.5 mg/m^2^	Not specified	0%	80%	6		0%
2018	Falkenstein [98]	7/5/1/0 (13)	2	20	69%	cisplatin 7.5 mg/m^2^ and doxorubicin 1.5 mg/m^2^ or oxaliplatin 92 mg/m^2^	None	15.4%	80%	2.8	0%	0%
2020	Di Giorgio [99]	9/4/3/4 (20)	2	18	60%	cisplatin 7.5 mg/m^2^ and doxorubicin 1.5 mg/m^2^ or oxaliplatin 92 mg/m^2^	Not specified	0%	50%	10	0%	0%
2021	Nielsen [100]	3/7/1/5 (16)	3			cisplatin 7.5 mg/m^2^ and doxorubicin 1.5 mg/m^2^ or oxaliplatin 92 mg/m^2^	Not specified	0%	61%	9.9		

PCI = Peritoneal cancer index, PRGS = Peritoneal regression grading score, OS = Overall survival, CRS = Cytoreductive surgery, CTCAE = Common terminology criteria for adverse events, CD = Clavien–Dindo.
